# Disrupting Pathogenicity in Foodborne *Staphylococcus aureus*: Biofilm Inhibition and Attenuation of Resistance and Virulence by Tunisian Aromatic Plant Essential Oils

**DOI:** 10.3390/foods15132361

**Published:** 2026-07-02

**Authors:** Amal Makhlouf, Hamouda Elabed, Sarra Moumni, Ameur Elaissi, Ahmed Reda Belmamoun, Khaloud Mohammed Alarjani, Lamia Hila, Abderrahmen Merghni

**Affiliations:** 1 Laboratory of Antimicrobial Resistance LR99ES09, Faculty of Medicine of Tunis, University of Tunis El Manar, Tunis 1007, Tunisia; 2 Department of Clinical Biology B, Faculty of Pharmacy of Monastir Tunisia, University of Monastir, Monastir 5000, Tunisia; 3 Laboratory of Pharmaceutical, Chemical and Pharmacological Drug Development LR12ES09, Faculty of Pharmacy, University of Monastir, Monastir 5000, Tunisia; 4 Process Engineering, Materials and Environment Laboratory, Faculty of Technology, Djillali Liabes University of Sidi Bel Abbes, Sidi Bel Abbès 22000, Algeria; 5 Department of Botany and Microbiology, College of Science, King Saud University, Riyadh 11451, Saudi Arabia; 6 Department of Genetic, Faculty of Medicine of Tunis, Tunis El Manar University, Tunis 1007, Tunisia

**Keywords:** food safety, essential oils, MRSA, GC-MS, antibacterial, antibiofilm, gene expression

## Abstract

The proliferation of methicillin-resistant *Staphylococcus aureus* (MRSA) in food processing is an escalating public health issue. This circumstance has intensified the quest for ecological alternatives to impede pathogen proliferation and avert food degradation. This study firstly investigated the chemical compositions of three essential oils (EOs) sourced from Eucalyptus, Rosemary and Lavender plants using GC-MS. Subsequently, the antibacterial and antibiofilm activities of the tested EOs were assessed against MRSA strains. The effects of these EOs on the expression of antibiotic resistance-related (*mecA*), virulence regulatory (*agrA* and *sarA*), and enterotoxin (*sea*) genes in MRSA strains were also evaluated by real-time PCR. Concerning the composition analyses performed on the EOs, our results revealed a total of 82 compounds, which accounted for 99.20, 98.10 and 92.78% of Eucalyptus, Rosemary and Lavender EOs, respectively. The anti-staphylococcal activity showed that Eucalyptus EO had the greatest effect, with diameter of inhibition exceeding 41 mm. Moreover, the association between Rosemary EO and the antibiotic (cefoxitin) highlighted the enhancement of the antibacterial effect against the MRSA reference strain. Additionally, Eucalyptus EO showed the highest inhibitory effect against both strains, with MIC values ranging from 0.781 to 1.563 mg/mL, followed by the Rosemary and Lavender EOs. All the tested EOs displayed a bactericidal effect against the tested MRSA strains. Regarding the antibiofilm activity, Rosemary and Lavender EOs had varying impacts on the pre-formed biofilms, with percentage reduction values ranging from 36% to 73% and 37% to 68%, respectively. Finally, the mRNA expression of the MRSA gene A *mecA* and virulence genes *agrA*, *sarA* and *sea* declined following EO treatment compared with the control. The findings of this study highlighted the efficacy of locally tested EOs in reducing MRSA biofilm formation and the expression of virulence factors and suggested their potential use in food safety and culinary applications.

## 1. Introduction

Methicillin-resistant *Staphylococcus aureus* (MRSA) infections have become significantly more common in community settings in recent years [1]. Recently, MRSA has been a significant and serious threat among pathogenic bacteria, and it has been accountable for almost 100,000 deaths [2]. With a case fatality rate between 15% and 30%, MRSA is the most common bacterial cause of global mortality [3]. Skin and subcutaneous tissue infections, pneumonia, bacteremia and food poisoning are only a few of the ailments caused by this opportunistic pathogen [2]. MRSA usually overcomes the effects of beta-lactams by producing penicillinase and altering the binding pocket for cell wall synthesis [3]. Furthermore, anti-glycopeptide medication resistance has started to emerge in the more current form of MRSA, which has made treating the infection difficult [4].

Alongside conventional resistance mechanisms, a distinctive characteristic of *S. aureus* pathogenesis is its capacity to endure on both living and non-living surfaces in a biofilm condition [5]. The creation of biofilm protects this bacterium against host immune system attacks, antibiotics, and external threats [6]. Additionally, a significant aspect in the pathogenic success of *S. aureus* is its array of virulence determinants, including secreted toxins, exoenzymes, and cofactors that activate host zymogens [7]. Taken together, all of these factors result in host tissue damage and the manipulation of its immune responses.

The investigation of innovative alternative antimicrobials, including natural essential oils (EOs), is receiving more attention due to the drawbacks of traditional antibiotics. EOs are being increasingly studied as natural, biodegradable antimicrobials to address multidrug-resistant bacteria and substitute synthetic agents [8]. Their abundance in chemicals such as 1.8 cineol, menthol, thymol, eugenol, carvacrol and cinnamaldehyde eradicates microorganisms by compromising cell membranes and subsequently restricting the development of bacterial resistance [9]. However, it was previously highlighted that the chemical compositions of EOs vary markedly according to various internal and external factors such as environmental and climatic conditions, as plants modify their secondary metabolism to adapt to stress [10,11]. Subsequently the variations in these factors affect the concentrations of terpenes and terpenoids in EOs, which, in turn, can impact or change their biological properties, notably their antimicrobial effects.

Although the antibacterial activities of EOs are well described, their modes of action in bacterial cells, particularly in pathogenic factor gene expression, need further investigation to better enable their valorization. Thus, our study aimed firstly to analyze the chemical compositions of local *Lamiaceae* and *Myrtaceae* species’ EOs, then to determine their antibacterial and antibiofilm activities against MRSA strains and finally to assess their effects in MRSA pathogenic factor gene expression.

## 2. Materials and Methods

### 2.1. Essential Oils and Chemical Composition

Three essential oils from two plant families, Lamiaceae (*Rosmarinus officinalis*; *Lavandula officinalis*) and Myrtaceae (*Eucalyptus globulus*), were purchased from a local producer (Herbéos, Khniss, Tunisia). Gas chromatography–mass spectrometry (GC–MS) and a gas chromatography–flame ionization detector (GC–FID) were used to investigate the essential oils’ chemical compositions [12].

### 2.2. Bacterial Strains

One biofilm forming the MRSA strain Sa12 [13] and one biofilm forming the MRSA reference strain NCTC12493 were used in this study for antibacterial and antibiofilm activities and for the assessment of the EOs’ effects in pathogenic factor gene expression.

### 2.3. Disk Diffusion Test

The antagonistic effects of *R. officinalis*, *L. officinalis* and *E. globulus* EOs were assessed against the two strains using the disk diffusion approach, as previously described [14]. The results were determined as the inhibitory zones (mm) surrounding EO-impregnated disks. As positive controls, cefoxitin (Fox 30 µg) disks (Oxoid, Thermo Fisher, Basingstoke, UK) were used.

### 2.4. Combined Disk Diffusion Test

For all of the MRSA strains, a combined disk diffusion assay was used to investigate potential interactions between the three EOs and cefoxitin. A Petri plate filled with Mueller–Hinton (MH) agar (Biolife, Milan, Italy) was covered with a bacterial inoculum suspension of each MRSA strain prepared in saline solution, achieving a turbidity of 0.5 McFarland. Then, cefoxitin (Oxoid, Dardilly, France) disks were impregnated with 10 μL of each EO and placed on the surfaces of the MH agar plates. After incubation for 24 h at 37 °C, the inhibition zones were determined, as mentioned in Section 2.2.

### 2.5. Microdilution Assay

The minimal inhibition concentration (MIC) and the minimal bactericidal concentration (MBC) of *R. officinalis*, *L. officinalis* and *E. globulus* EOs were assessed against the two MRSA strains, as previously described [15]. A range of concentrations of the studied substances, varying from 50 to 0.098 mg/mL, was prepared by performing serial twofold dilutions with dimethyl sulfoxide (DMSO), followed by dilution in MH broth.

### 2.6. Antibiofilm Activities

#### 2.6.1. Biofilm Inhibition

The biofilm-inhibiting properties of the selected EOs were assessed as previously described [16]. Sub-inhibitory concentrations (1/8 to 1 × MIC) of the investigated substances were applied to each bacterial strain formerly cultured in BHI (with 2% glucose). Non-adherent cells were eliminated following a 24 h incubation at 37 °C, and biofilm cells dyed with Crystal Violet (1%) were measured at 570 nm. Wells containing bacterial inoculum without essential oils were used as the positive control, whereas wells with BHI broth only were reserved for the negative control.

#### 2.6.2. Biofilm Eradication

*R. officinalis*, *L. officinalis* and *E. globulus* EOs’ capacity to eradicate biofilms was evaluated in accordance with earlier reports [16]. The selected EOs were added to pre-established biofilms (48 h) at different concentrations, ranging from MIC to 4 × MIC, and then incubated for an additional 24 h. CV (1%) was used to stain the treated biofilm biomass, and its absorbance at 570 nm was used to measure the results. The following formula was used to estimate the percentage of biofilm eradication:

[(OD growth control − OD sample)/OD growth control] × 100

### 2.7. Evaluation of Virulence Gene Expression by Quantitative Real-Time PCR

#### 2.7.1. Bacterial RNA Extraction

After the overnight treatment of clinical and MRSA strains with MIC of *R. officinalis*, *L. officinalis* and *E. globulus* EOs, the total RNA from each bacterial strain was isolated using the FavorPrep™ Tissue Total RNA MicroElute Kit (Favorgen, Tainan, China), following the manufacturer’s protocol adapted for bacterial samples. The extracted RNA was treated with DNase I to remove residual genomic DNA. RNA quality and concentration were assessed using a NanoDrop 2000 spectrophotometer (Thermo Scientific, Waltham, MA, USA).

#### 2.7.2. Quantitative RT-PCR Assay

Reverse transcription was performed at a final volume of 20 μL containing 100 ng of RNA, 1 mM of dNTP mix, 100 ng of random hexamers, 4 μL of 5 × SCRIPT RT buffer, and 200 U of SCRIPT Reverse Transcriptase (Jena Bioscience, Thuringia, Germany; Cat. No. PCR-505L). The reaction was incubated for 20 min at 42 °C, followed by 30 min at 50 °C, and terminated by heating at 70 °C for 10 min. The resulting cDNA was subjected to quantitative real-time PCR using the Rotor-Gene Q MDx system (Qiagen, Hilden, Germany). Each sample was analyzed in triplicate in a 20 μL reaction containing 10 μL of qPCR GreenMaster (Jena Bioscience; Cat. No. PCR-372L), 2 μL of cDNA, 1 μL of each primer (10 μM), and 6 μL of PCR-grade water. The amplification conditions were 95 °C for 2 min, followed by 40 cycles of 95 °C for 20 s and 60 °C for 60 s. Cycle threshold (Ct) values were determined using the Rotor-Gene Q Series Software v2.0. PCR specificity was confirmed by high-resolution melting (HRM) analysis by increasing the temperature from 50 °C to 95 °C. The relative expression of virulence factor genes was calculated according to the Pfaffl method [17]. Primer sequences are listed in Table 1, and normalization was performed using the *16S rRNA* gene.

### 2.8. Statistical Analysis

All experiments were performed in triplicate, and the results are shown as the mean values ± standard deviations. The average values were calculated using the SPSS 25.0 statistical package for Windows. A significance test was conducted on the treatments using a two-way ANOVA. Differences in means were calculated using Duncan’s multiple range tests for means with 95% confidence intervals (*p* ≤ 0.05).

## 3. Results

### 3.1. Chemical Composition of the Essential Oils

GC-MS-based chemical composition analyses of the studied EOs revealed a total of 82 compounds, as reported in Table 2. They accounted for 99.20, 98.10 and 92.78% of Eucalyptus, Rosemary and Lavender EOs. 1.8-cineol (83.4%) and α-pinene (8.2%) were the major components of Eucalyptus essential oil. Regarding Rosemary EO, the main components were 1.8-cineol (46.9%), L-camphor (13.1%) and α-pinene (12.36%). For Lavender EO, Linalool (35.7%) and Linalyl acetate (33.4%) were found to be the main components.

### 3.2. Disk Diffusion Susceptibility Test

The antibacterial effects of the selected EOs were firstly assessed by the disk diffusion assay against the clinical and reference strains of MRSA, both alone and combined with the antibiotic cefoxitin. Our results revealed that the Eucalyptus EO exhibited the highest effect, with a diameter of inhibition exceeding 41 mm. Moreover, the association between the Rosemary EO and the antibiotic showed the enhancement of the antibacterial effect against the reference strain (18.50 mm) compared to when the EO was tested alone (16.67). A slight amelioration of this activity was also registered with the Lavender and Eucalyptus EOs against the same tested strain (Table 3); however, no positive effect of this association (EO/ATB) was noted against the clinical strain Sa12.

### 3.3. MIC and MBC Determination

The results for the antibacterial effects of the selected EOs against the MRSA strains are summarized in Table 4. The Eucalyptus EO showed the highest inhibitory effect against both strains, with MIC values ranging from 0.781 to 1.563 mg/mL, followed by the Rosemary EO and the Lavender EO. Additionally, the obtained results for the MBCs of the investigated oils range from 1.563 to 6.250 mg/mL. Taken together, all the tested EOs exhibited a bactericidal effect against MRSA bacteria, with MBC/MIC values ≤ 4.

### 3.4. Anti-Adhesion Effect

**Figure 1:**
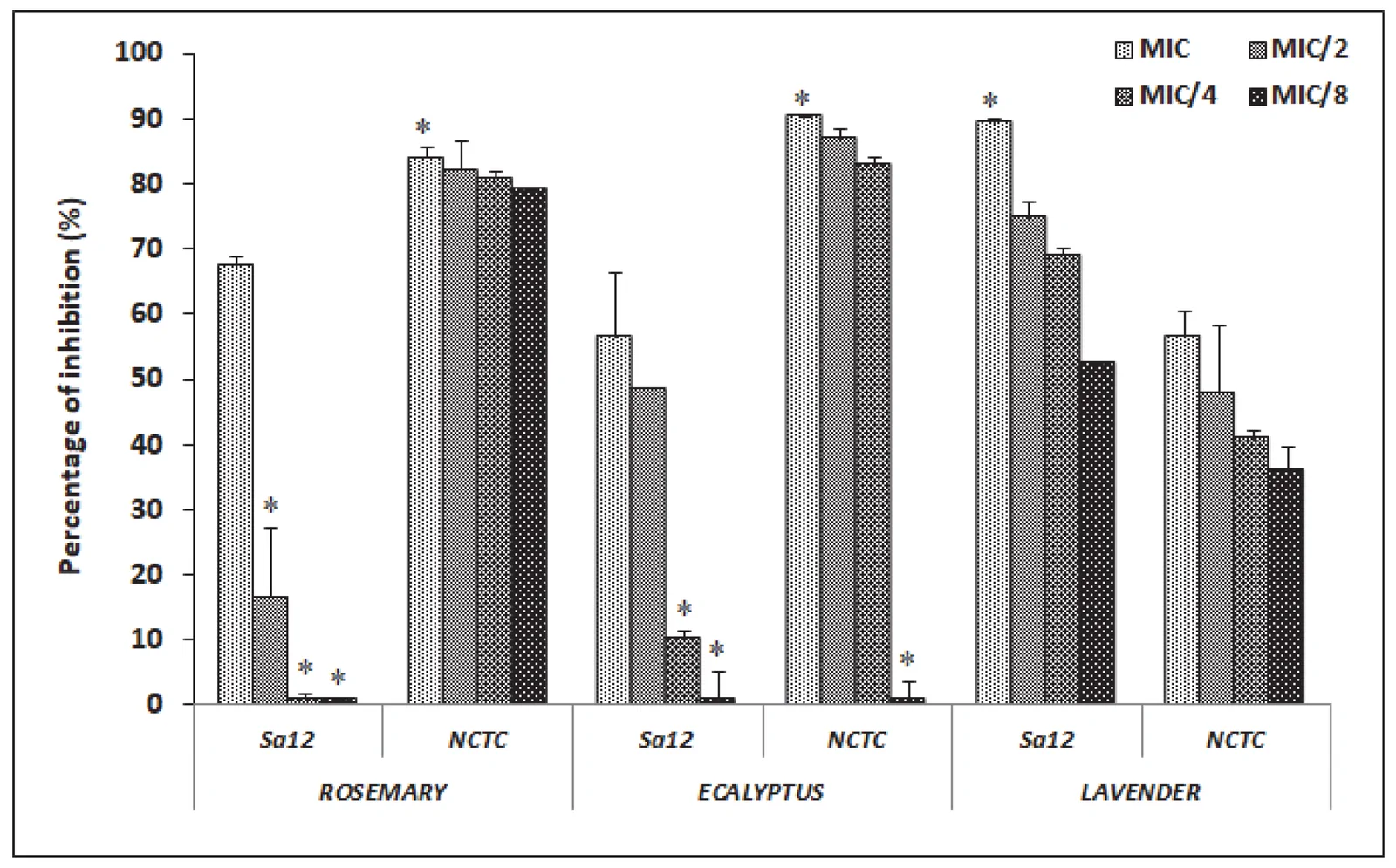
The effects of the Eucalyptus, Rosemary and Lavender essential oils on the adhesion of the clinical (Sa12) and reference (NCTC12493) MRSA strains treated with sub-inhibitory concentrations (1/8 to 1 × MIC), expressed as a percentage of inhibition (%) and evaluated by the Crystal Violet staining assay. Values are the averages of at least three independent determinations. Error bars represent standard deviations. (*) Differences were considered significant at *p* < 0.05.

Following bacterial incubation for 24 h with sub-inhibitory concentrations of the investigated agents, the pre-formed biofilm was stained with Crystal Violet, and the percentage of anti-adhesion activity was determined after comparison with untreated bacteria. The Rosemary and Eucalyptus EOs were more effective against the reference strain, with the percentage of biofilm inhibition ranging from 71% to 93%. However, the Lavender EO showed a potent anti-attachment effect against the clinical MRSA strain, with the percentage of inhibition exceeding 52% at a low dose (1/8 MIC) (Figure 1).

### 3.5. Biofilm Eradication Effect

**Figure 2:**
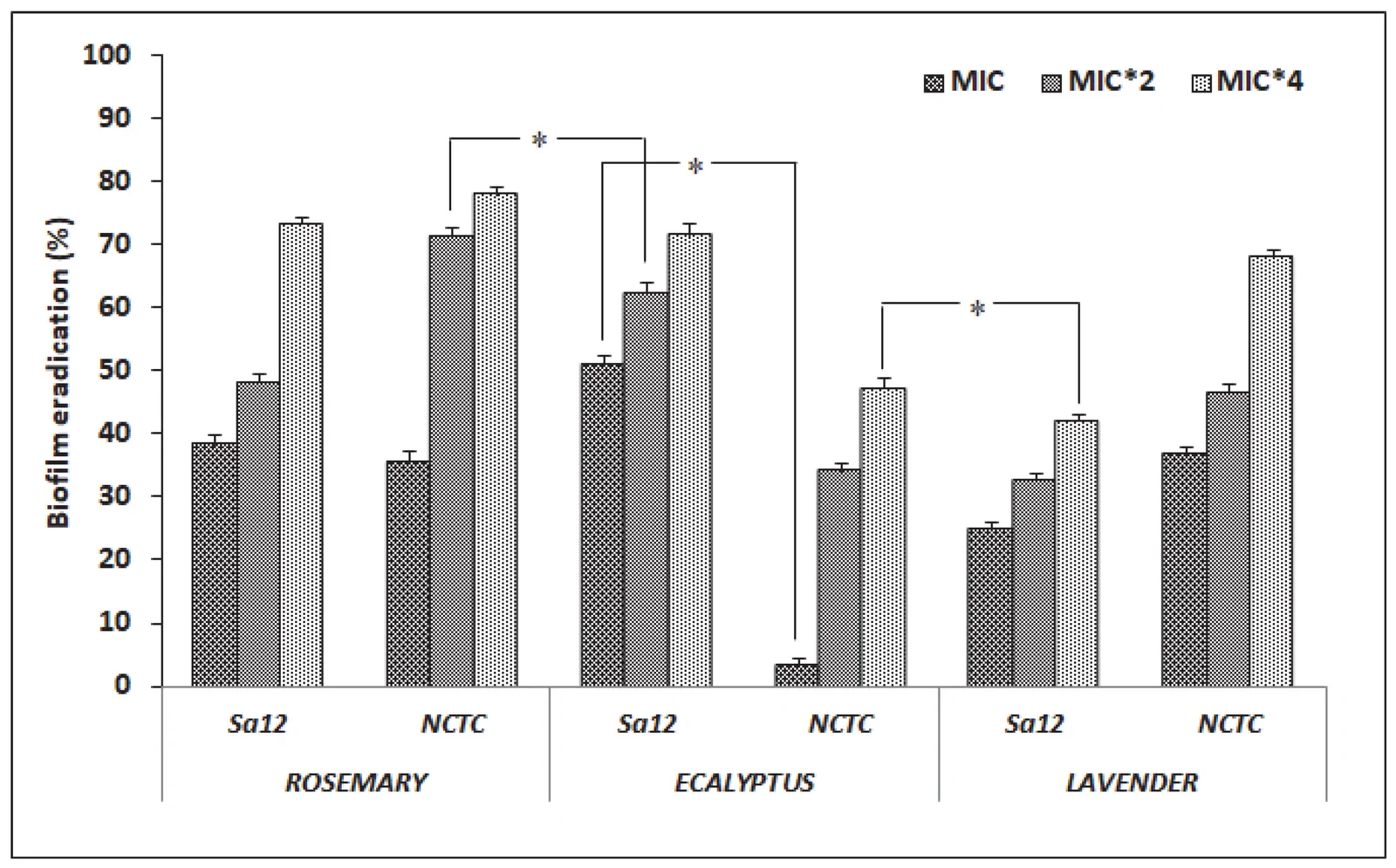
The effects of the Eucalyptus, Rosemary and Lavender essential oils on pre-formed biofilms of the clinical (Sa12) and reference (NCTC12493) MRSA strains treated with various concentrations (MIC to MIC × 4), expressed as a percentage of biofilm eradication (%) and evaluated by the Crystal Violet staining assay. Values are the averages of at least three independent determinations. Error bars represent standard deviations. (*) Differences were considered significant at *p* < 0.05.

MRSA strains’ biofilms were treated with the selected EOs at different concentrations ranging from MIC to 4 × MIC. The results of this test are presented in Figure 2. The Rosemary and Lavender EOs had varying impacts on pre-formed biofilms, with percentage reduction values ranging from 36% to 73% and 37% to 68%, respectively (Figure 2). In contrast, the Eucalyptus EO was more efficient against the clinical MRSA biofilm, with the percentage of eradication exceeding 50%, even at low concentration (1 × MIC).

### 3.6. Pathogenic Factor Gene Expression

**Figure 3:**
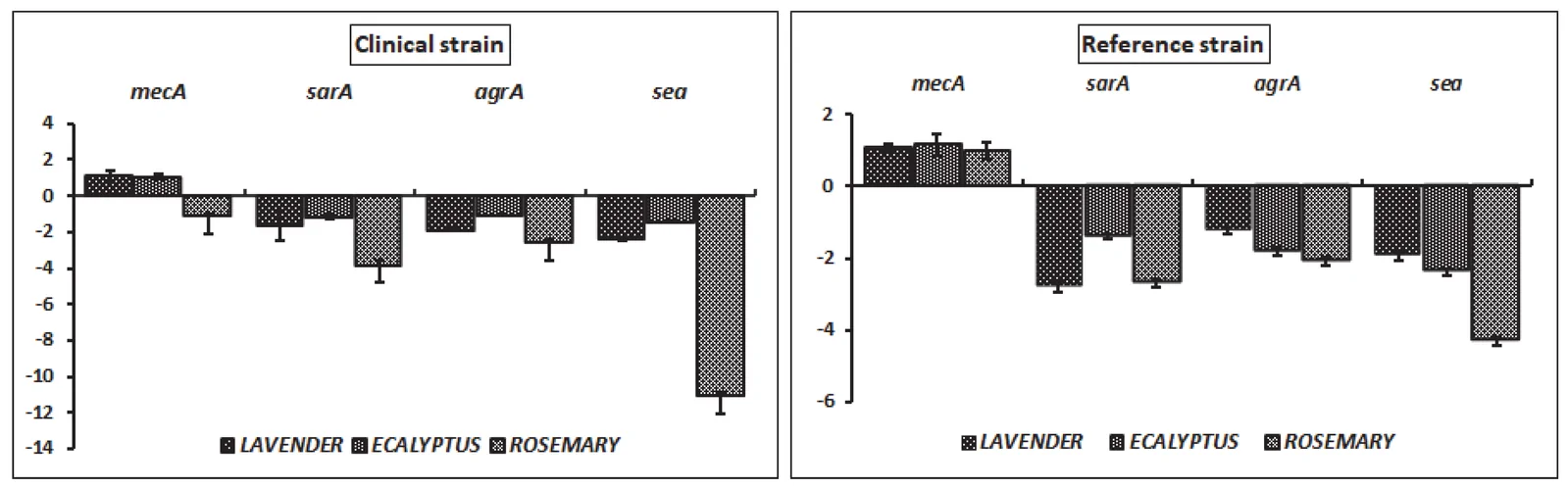
The relative expression levels (fold change) of antibiotic resistance (*mecA*) and virulence (*sarA*, *agrA*, and *sea*) genes in clinical (Sa12) and reference (NCTC12493) *MRSA* strains following exposure to Lavender, Eucalyptus, and Rosemary essential oils. Gene expression was quantified by real-time quantitative PCR (RT-qPCR) and normalized to the untreated control condition. Fold change values represent relative transcription levels calculated using the 2^−ΔΔCt^ method. Negative values indicate down-regulation, whereas positive values indicate the upregulation of gene expression compared to the control. Among the tested treatments, the Rosemary essential oil induced the strongest down-regulation of virulence-associated genes, particularly *sea* and *sarA*, with a more pronounced effect observed in the clinical strain compared to the reference strain.

By analyzing the mRNA expression of the *mecA*, *agrA*, *sarA*, and *sea* genes in MRSA strains, we evaluated the impacts of the EOs on virulence-related gene expression and antibiotic resistance. Treated strains were subjected to MICs of each EO, and the data presented in Figure 3 demonstrate the suppression of gene expression for all target genes in comparison to untreated bacterial cells.

Quantitative real-time PCR analysis revealed variable gene expression since *mecA* expression in the reference MRSA strain remained unaffected or slightly upregulated, with fold changes of +1.07, +1.14 and 1.00 for Lavender, Eucalyptus, and Rosemary EO treatments, respectively (Figure 3). In contrast, consistent down-regulation was observed for the regulatory genes *sarA* and *agrA*, with the strongest inhibition induced by Rosemary oil (−2.72 and −2.08, respectively), followed by Lavender (−2.81 and −1.22) and Eucalyptus (−1.40 and −1.82) oils. The *sea* gene exhibited the most pronounced repression, particularly under Rosemary treatment (−4.33), compared to Eucalyptus (−2.38) and Lavender (−1.95). Regarding the Sa12 strain, a different expression pattern was observed, characterized by a greater sensitivity to essential oil exposure. Notably, consistent down-regulation of *sarA* and *agrA* was detected, with fold changes of −3.79 and −2.54, respectively, indicating a particularly strong inhibitory effect from Rosemary oil. Remarkably, the *sea* gene exhibited a dramatic reduction in expression in this clinical MRSA strain in the presence of Rosemary oil (−11.02) compared to other treatments.

## 4. Discussion

Essential oils are being extensively studied due to their abundant and varied bioactive constituents, which provide a natural, safe and effective option to address drug-resistant microorganisms. In the first part of our study, the chemical composition analyses of three local EOs (Eucalyptus, Rosemary and Lavender) performed by GC-MS revealed a total of 82 compounds pertaining to Monoterpene oxide (1.8-cineol), Monoterpene hydrocarbon (α-pinene), Monoterpene keton (L-camphor), Monoterpene alcool (Linalool), and Monoterpene ester (Linalyl acetate). Previous studies performing GC-MS analysis of the Lamiaceae (Rosemary and Lavender) and Myrtaceae (Eucalyptus) species’ EOs, studying species with different geographical origins (Morocco, France, Italy, Greece and Turkey), revealed the presence of the same main constituents but with difference percentage contents [19,20,21]. The variations in these compositions arise from the confluence of biological and genetic variables, environmental conditions and even the difference between the methodologies used for extraction and storage [22,23]. Additionally, differences in minor compounds of EOs have been described [24,25], and the profile compound fluctuations were registered even within the same plant organs [26,27]. All these fluctuations in EOs’ compositions certainly affected their biological activities, making the analysis of their constituents important to better understand their modes of action in pathogenic bacterial cells.

The antibacterial effects of the selected EOs were firstly assessed using a disk diffusion assay against clinical and reference strains of MRSA. Our results revealed that the Eucalyptus EO exhibited the greatest effect, with diameters of inhibition ranging between 38 and 41.33 mm. This important activity may be due to the high percentage of 1.8-cineol (83.4%) present in this analyzed EO. More specifically, it was previously reported that Eucalyptus (1.8-cineol) induces reactive oxygen species (ROS)-mediated oxidative stress and compromises the integrity of the cell membrane in MRSA strains [28]. In fact, the surge in ROS generation influenced antioxidant enzyme function and compromised macromolecules, resulting in bacterial damage and subsequent cell death [29]. Moreover, the association between the tested EOs and the antibiotic (Fox) showed the enhancement of the antibacterial effect against the reference strain; however, no positive effect of this association was noted against the clinical MRSA strain. Our results are in agreement with other reports showing a synergistic effect between EOs and antibiotics [30,31]. The observed differences may be related to the types of interactions of the minor and major compounds of EOs with the antibiotic and the susceptibility of the tested bacterial strain. The antibacterial effects of the selected EOs against the MRSA strains evaluated by the determination of MICs were found to be in agreement with those for the disk diffusion assay, since the Eucalyptus EO showed the greatest inhibitory effect against both strains, with MIC values ranging from 0.781 to 1.563 mg/mL, followed by the Rosemary EO and the Lavender EO. All tested EOs exhibited bactericidal effects against MRSA bacteria with MBC/MIC values ≤ 4 [32].

The roles of bacterial biofilms in antibiotic resistance, along with the different control strategies used, were studied in [33,34]. Our study investigated a biological biofilm control approach based on plant-extracted EOs. The percentages of anti-adhesion activity, determined through comparison with untreated bacteria strains, revealed that Rosemary and Eucalyptus EOs were more effective against the MRSA reference strains, with the percentage of biofilm inhibition reaching 93%. Regarding the Lavender EO, a potent anti-attachment effect was noted against the clinical MRSA strain at a low dose (1/8 MIC). Since initial adhesion is a crucial step in biofilm formation [35], it is of interest to impede this phase and subsequently to prevent biofilm development and maturation. In agreement with our study, it was revealed that EOs are effective natural agents for initial biofilm suppression [36,37]. In addition to biofilm attachment inhibition, we investigated the biofilm eradication potency of the selected EOs. Our results showed that the Eucalyptol EO was more efficient against the clinical MRSA biofilm, with the percentage of eradication exceeding 50% even at low concentrations (1 × MIC). Regarding the Rosemary and Lavender EOs, varying effects on pre-formed biofilms formation were observed, with percentage reduction values ranging from 36% to 73%. Accordingly, plant-derived bioactive chemicals, exemplified by those in Eucalyptus, Rosemary and Lavender, may proficiently destroy biofilm architectures by processes including quorum-sensing (QS) inhibition [15,38,39]. In fact, the QS mechanism acts as an intercellular signaling system through chemical signals, enabling bacterial communication and control of their biofilm formation [40]. Therefore, EOs function as natural anti-quorum-sensing agents against MRSA by interrupting bacterial communication, which consequently prevents biofilm formation and virulence factor generation [41,42].

The last part of our study was conducted to assess the effects of local EOs on methicillin resistance and virulence-related gene expression in both MRSA strains by quantitative PCR. This comparative analysis highlights a more pronounced transcriptional response in the clinical Sa12 strain, particularly for *sea* and *sarA* genes, suggesting increased susceptibility to EO-mediated regulation. Among the tested oils, Rosemary consistently exerted the strongest inhibitory effect on pathogenicity-related gene expression in both strains, followed by Eucalyptus and Lavender. Remarkably, upregulated expression of the antibiotic resistance gene *mecA* was noted following treatment with the majority of EOs. Our findings were in agreement with a recent report revealing that the mRNA expression of pivotal genes implicated in MRSA resistance and pathogenicity declined in a concentration-dependent manner following *Chrysanthemum zawadskii* EO treatment compared to untreated bacteria [18]. Overall, these findings indicate that essential oils, especially Rosemary, significantly repress key virulence determinants in *MRSA* in a strain-dependent manner, potentially through interference with global regulatory systems such as *sarA* and *agrA*.

## 5. Conclusions

The present study highlighted the variety and abundance of chemical components present in local essential oils from *Lamiaceae* and *Myrtaceae* species. This diversity in bioactive constituents justifies the observed and notable antibacterial, anti-adhesion and antibiofilm properties of these EOs against MRSA pathogenic strains. Additionally, our study revealed that the tested EOs inhibited the expression of methicillin resistance, staphylococcal enterotoxin A, staphylococcal accessory regulator A and accessory gene regulator A, which are considered major genes linked to the resistance and pathogenicity of MRSA. Accordingly, this molecular targeting approach requires further investigation for potential use in food applications aiming at lowering MRSA’s virulence and infectivity.

## Figures and Tables

**Table 1:** The primers used in the RT-PCR experiments [18].

Gene	Sequences (5′ → 3′)		Tm (°C)	Base Count (nt)	GC Ratio (%)
*agrA*	Forward	TGATAATCCTTATGAGGTGCTT	53.7	22	36.36
	Reverse	TGATAATCCTTATGAGGTGCTT	55.6	21	42.86
*mecA*	Forward	GTTAGATTGGGATCATAGCGTCATT	58.1	25	40
	Reverse	TGCCTACTCATGTGTTCCTGTAT	59	27	37.04
*sarA*	Forward	TGTTATCAATGGTCACTTATGCTG	56.3	24	37.5
	Reverse	TCTTTGTTTTCGCTGATGTATGTC	57.1	24	37.5
*sea*	Forward	ATGGTGCTTATTAGGTGTATC	50.1	21	33.33
	Reverse	CGTTTCCAAAGGTAGTGTTATT	52.9	21	38.1
*16s rRNA*	Forward	ACTGGGATAACTTCGGGAAA	55.2	20	45
	Reverse	CGTTGCCTTGGTAAGCC	54.9	17	58.82

**Table 2:** The chemical compositions of the tested EOs.

N°	TR	IR	Compounds	Rosemary	Eucalyptus	Lavender
1	3.561	852	Cis-3-hexenol	0.01	*	Tr
2	4.556	910	Zeta-fenchene	0.02	*	*
3	4.537	917	Alpha-fenchene	*	0.18	*
4	4.946	925	Tricyclène	0.11	*	0.01
5	5.043	928	Alpha-Thujène	0.14	tr	0.03
6	5.227	936	Alpha-pinene	12.36	8.20	0.77
7	5.579	949	Camphéne	2.92	0.06	0.15
8	5.718	954	Verbenene	0.05	*	0.01
9	6.230	974	Sabinene	0.21	*	0.12
10	6.330	978	Beta-Pinène	5.83	0.45	0.71
11	6.5347	986	3-octanone	*	*	0.05
12	6.717	993	Myrcène	1.53	0.15	0.37
13	6.8252	997	Butyl butyrate	*	*	0.03
14	7.132	1006	Alpha-Phellandrene	0.22	0.11	0.05
15	7.329	1012	Delta-3-Carene	0.19	*	0.07
16	7.3766	1013	Hexyl acetate	*	*	0.02
17	7.529	1017	Alpha-Terpinene	0.13	0.02	0.02
18	7.787	1024	P-Cymène	3.52	2.29	0.58
19	8.087	1033	1,8-cinéole	46.89	83.40	5.55
20	7.9270	1028	Limonène	*	*	0.55
21	8.246	1037	Trans-beta-Ocimene	0.09	*	0.24
22	8.3451	1040	N-Butyl isovalerate	*	*	0.02
23	8.599	1047	Cis-beta-Ocimene	0.06	*	0.11
24	8.969	1057	Gamma-Terpinene	0.55	0.06	0.14
25	9.454	1070	Cis-Sabinene hydrate	0.05	*	*
26	9.2614	1065	Trans-sabinene hydrate	*	*	0.10
27	9.383	1068	Cis-Linalool oxide	*	0.17	*
28	9.4566	1070	Trans-Linalool oxide	*	*	0.16
29	10.020	1086	P-alpha-dimethylstyrene	*	0.42	*
30	10.065	1087	Alpha-Terpinolene	0.22	0.27	0.20
31	10.353	1095	Terpinolene	*	0.08	*
32	10.510	1100	Linalool	0.52	0.03	35.68
33	10.662	1103	Isoamyl isovalerate	0.02	0.07	*
34	11.0462	1112	Cis-rose oxide	*	*	0.16
35	11.349	1119	Fenchol	0.05	*	*
36	11.499	1123	Campholenal	0.05	0.02	*
37	11.5093	1123	Chrysanthenone	*	*	0.18
38	11.964	1134	Trans-Pinocarveol	*	2.36	*
39	12.254	1141	L-camphor	13.12	0.07	4.46
40	12.421	1145	Exo-methyl-camphenilol	0.07	*	*
41	12.5184	1147	Hexyl isobutyrate	*	*	0.11
42	12.7836	1153	Isoborneol	*	*	0.60
43	12.982	1158	Pinocarvone	0.03	0.05	*
44	13.160	1162	Borneol L.	2.27	0.12	3.53
45	13.377	1167	Isopinocamphone	*	0.13	*
46	13.642	1173	Terpinene-4-ol	0.48	0.12	2.21
47	13.903	1180	P-cymen-8-ol	*	0.06	*
48	14.026	1182	Is-p-Mentha-1(7),8-dien-2-ol	*	0.12	*
49	14.220	1187	Alpha-Terpineol	1.34		0.52
50	14.530	1194	Myrtenol	0.12	0.11	0.04
51	14.687	1198	Methyl chavicol	0.03	*	*
52	14.9891	1205	Verbenone	*	0.03	0.04
53	15.2214	1210	Octyl acetate	*	*	0.02
54	15.446	1215	Trans-(+)-carveol	0.04	0.04	0.02
55	15.802	1223	Bornyl formate	0.03	*	*
56	15.8028	1223	Isobornyl formate	*	*	0.03
57	15.9318	1226	Nerol	*	*	0.08
58	16.5292	1240	Hexyl isovalerate	*	*	0.13
59	17.3515	1258	Linalyl acetate	*	*	33.40
60	18.368	1281	Bornyle acetate	0.73	0.02	0.03
61	18.7064	1289	Lavandulyl acetate	*	*	1.44
62	18.954	1294	Thymol	0.07	*	*
63	19.098	1297	Carvacrol	0.02	0.02	*
64	21.214	1345	Alpha-cubebene	0.06	*	*
65	21.484	1351	Eugenol	0.09	*	*
66	22.136	1366	Alpha-yalangene	0.10	*	*
67	22.332	1370	Alpha-Copaene	0.20	*	*
68	22.999	1385	Beta-Elemene	0.05	*	*
69	23.576	1398	Methyl eugenol	0.08	*	*
70	24.169	1412	Beta-Caryophyllene	1.95	*	*
71	24.568	1422	Alpha cedrene	0.07	*	*
72	24.990	1431	Aromadendrene	0.07	*	0.04
73	25.603	1446	Alpha-Humulène	0.26	*	*
74	25.878	1452	(E)-beta-farnesene	0.05	*	*
75	26.619	1470	Alloaromadendrene	0.17	*	*
76	26.766	1473	Germacrene D	0.02	*	*
77	27.374	1487	Ar-Curcumene	0.08	*	*
78	27.624	1493	Beta-selinene	0.09	*	*
79	28.010	1502	Beta-bisabolene	0.06	*	*
80	28.159	1506	Alpha-amorphene	0.11	*	*
81	28.568	1516	Delta-cadinene	0.22	*	*
82	30.878	1574	Caryophyllene oxide	0.26	*	*
Total (%)				98.10	99.20	92.78

*: Trace (<0.1%).

**Table 3:** The disk diffusion assay.

EO	MRSA	D.I (mm)	ATB (Fox)	EO + ATB
Rosemary	Sa12	19.67 ± 0.58	6.00	19.33 ± 0.58
	NCTC12493	16.67 ± 1.15	6.00	18.50 ± 1.15
Eucalyptus	Sa12	41.33 ± 1.15 *	6.00	34.67 ± 1.15 **
	NCTC12493	38.00 *	6.00	38.67 ± 0.58 **
Lavender	Sa12	18.00	6.00	18.00
	NCTC12493	19.67 ± 0.58	6.00	20.50 ± 0.58

D.I: Diameter of inhibition; ATB: antibiotic; FOX: cefoxitin; * indicates a statistically significant difference between the activity of the EOs tested without the antibiotic; ** indicates a statistically significant difference between the activity of the EOs tested in association with the antibiotic.

**Table 4:** The determination of the MICs and MBCs of the EOs.

EO	Sa12			NCTC12493		
	MIC	MBC	MBC/MIC	MIC	MBC	MBC/MIC
Rosemary	1.563	3.125	2	3.125	6.250	2
Eucalyptus	0.781	1.563	2	1.563	1.563	1
Lavender	3.125	6.250	2	6.250	6.250	1

Sa12: clinical MRSA strain; NCTC12493: reference MRSA strain; MIC: minimal inhibition concentration; MBC: minimal bactericidal concentration. MIC and MBC values are expressed as mg/mL.

## Data Availability

The original contributions presented in this study are included in the article. Further inquiries can be directed to the corresponding author.
